# Hospital admission risk stratification of patients with gout presenting to the emergency department

**DOI:** 10.1007/s10067-021-05902-5

**Published:** 2022-01-01

**Authors:** Wang Han, Nur Azizah Allameen, Irwani Ibrahim, Preeti Dhanasekaran, Feng Mengling, Manjari Lahiri

**Affiliations:** 1 Saw Swee Hock School of Public Health, National University Health System, National University of Singapore, 12 Science Drive 2, Singapore, 117549 Singapore; 2 Division of Rheumatology, Department of Medicine, National University Hospital, Singapore, Singapore; 3 Emergency Medicine Department, National University Hospital, National University Health System, Singapore, Singapore; 4 Department of Surgery, Yong Loo Lin School of Medicine, National University of Singapore, Singapore, Singapore; 5 Department of Medicine, Yong Loo Lin School of Medicine, National University of Singapore, Singapore, Singapore; 6 Institute of Data Science, National University of Singapore, Singapore, Singapore

**Keywords:** Clinical decision support systems, Hospital, Emergency service, Gout

## Abstract

**Abstract:**

To characterise gout patients at high risk of hospitalisation and to develop a web-based prognostic model to predict the likelihood of gout-related hospital admissions. This was a retrospective single-centre study of 1417 patients presenting to the emergency department (ED) with a gout flare between 2015 and 2017 with a 1-year look-back period. The dataset was randomly divided, with 80% forming the derivation and the remaining forming the validation cohort. A multivariable logistic regression model was used to determine the likelihood of hospitalisation from a gout flare in the derivation cohort. The coefficients for the variables with statistically significant adjusted odds ratios were used for the development of a web-based hospitalisation risk estimator. The performance of this risk estimator model was assessed via the area under the receiver operating characteristic curve (AUROC), calibration plot, and brier score. Patients who were hospitalised with gout tended to be older, less likely male, more likely to have had a previous hospital stay with an inpatient primary diagnosis of gout, or a previous ED visit for gout, less likely to have been prescribed standby acute gout therapy, and had a significant burden of comorbidities. In the multivariable-adjusted analyses, previous hospitalisation for gout was associated with the highest odds of gout-related admission. Early identification of patients with a high likelihood of gout-related hospitalisation using our web-based validated risk estimator model may assist to target resources to the highest risk individuals, reducing the frequency of gout-related admissions and improving the overall health-related quality of life in the long term.

****Key points**:**

• *We reported the characteristics of gout patients visiting a tertiary hospital in Singapore.*

• *We developed a web-based prognostic model with non-invasive variables to predict the likelihood of gout-relatedhospital admissions.*

**Supplementary Information:**

The online version contains supplementary material available at 10.1007/s10067-021-05902-5.

## Introduction

Gout is a common chronic crystal deposition disorder with a worldwide prevalence ranging from < 1 to 6.8% and an incidence of 0.58–2.89 per 1,000 person-years [1]. A high prevalence of comorbidity, primarily hypertension, hyperlipidaemia, diabetes mellitus, and chronic kidney disease in individuals with gout is well-recognised [1–3].

Despite considerable advances in the understanding of the pathophysiology of gout and the availability of effective treatment, patients continue to have flares of gout requiring emergency department (ED) attendance and hospitalisation, constituting a significant healthcare and economic burden. Hospital admissions have increased by 50–100% in the UK and the USA over the past 15–30 years [4–6]. A nationwide US study demonstrated an increase in the ED charges from $195 million in 2009 to $287 million in 2012. New Zealand, with one of the highest reported prevalence rates of gout, reported a mean of 2.9 (SD 2.2) admissions per individual per year [2, 7]. These trends may be attributable to suboptimal long-term treatment of gout; however, recent studies have described the added contribution of older age and the presence of gout-associated comorbidities [7, 8].

Characterisation of a gout cohort with a high risk of hospitalisation may help to identify a target group for the development and early institution of targeted interventions to reduce the frequency of gout-related hospitalisation, and eventually, improvement of the overall health-related quality of life, of these complex multimorbid patients. We therefore performed this study to (a) identify the predictors of hospital admission for patients presenting to the ED for gout flares and (b) develop a prognostic model that may be used to predict the likelihood of gout-related hospital admissions.

## Materials and methods

We conducted a retrospective single-centre cohort study at a tertiary hospital in Singapore of patients presenting to the ED with a gout flare between 1 January 2015 and 30 September 2017. The patients were identified from the hospital’s electronic health record (EHR) system which links the ED attendances, patient’s history, previous admissions, laboratory tests, medications, and procedures using a unique identifier. The included patients were either diagnosed with gout flare during their ED visit or hospitalised within 3 days of their latest ED visit with a primary inpatient diagnosis of gout regardless of their primary ED diagnosis. Patients who were diagnosed with gout in ED and hospitalised within 3 days but had a primary inpatient diagnosis other than gout were excluded. For patients with multiple eligible ED visits, only one visit was chosen randomly and included in the final cohort to make sure the data were independent of each other. This study was approved by the Domain Specific Review Board (DSRB) (Ref: 2017/00975) and was granted a waiver of informed consent.

Patient demographics (age, gender, ethnicity); chronic comorbidities including hypertension, hyperlipidemia, and the ones in the Charlson Comorbidity Index [9]; and their past medical resource utilisation prior to the ED visit were retrieved from the hospital’s electronic medical records. For chronic comorbidities, we applied a minimum 1-year look-back period and extracted from all our accessible data since 1 Jan 2014 to capture as many as possible. We extracted the chronic comorbidities based on the ICD-9 and ICD-10 diagnosis codes from all previous visits of the patients and grouped them as shown in Supplementary Table 1. HIV was not included due to a prevalence of zero in our dataset. To validate the comprehensiveness of the coding, we also looked at medication prescription data and laboratory tests for diabetes and chronic kidney disease, which were two of the most common comorbidities in our patients. We considered the patient to have diabetes if (1) the prescription data contained at least one prescription for insulin or any oral hypoglycaemic agent, (2) there were at least two measurements of glycated haemoglobin (HbA1c) ≥ 6.5%, or (3) there were at least two measurements of random glucose > 11 mmol/L or fasting glucose > 7 mmol/L. We considered the patient to have chronic kidney disease if (1) there was a diagnosis or procedure code for dialysis or (2) there were at least two measurements of serum creatinine > 130 umol/L taken a minimum of 3 months apart. For medical resource utilisation, we applied an exact 1-year look-back period to only capture the most recent information of the patient. We defined patients with at least one prescription code for either allopurinol, febuxostat, or probenecid from previous outpatient, ED, or inpatient visits to be on urate-lowering therapy (ULT). We defined patients with at least one prescription code for either prednisolone, colchicine, non-steroidal anti-inflammatory drugs (NSAID), or COX-2 inhibitors (COXIB) from previous outpatient or ED visits to have stand by acute gout treatment (AGT). We also abstracted data on previous ED visits, outpatient visits with a primary diagnosis of gout, or previous hospitalisation with an inpatient primary diagnosis of gout. Severity of the gout flare and number or site of joints involved were unavailable in our dataset; hence, we extracted the ED foot, knee, ankle, wrist, elbow, and shoulder radiograph orders from the billing data and used them to create surrogate markers for (1) the severity of gout flare (whether the patient received an ED joint radiograph), (2) the total number of joints involved (number of joints that a radiograph was ordered for), and (3) the site of joints involved (whether the radiograph was ordered for upper or lower limb joints). Statistical analysis was conducted to compare patients who were hospitalised with those who were discharged from the ED (Kruskal–Wallis H test for continuous variables and Chi-squared test for categorical variables).

We split our dataset randomly, with 80% of cases used to form the derivation set and the remaining 20% used to form a validation set to validate our risk prediction model. A multivariable logistic regression model was used to ascertain the likelihood of hospitalisation from a gout flare in the derivation set. Adjusted odds ratios with 95% confidence intervals were calculated. Variables with statistically significant adjusted odds ratios were used to build a risk estimator for hospitalisation. We validated our model on the validation subset in three ways. (1) Area under the receiver operating characteristic curve (AUROC) was used to evaluate the performance of the model. (2) We drew the calibration plot by binning patients into deciles based on their predicted risk and showed the fraction of positive outcome in each decile versus the average predicted risk scores in each decile. The perfect calibration curve would be the diagonal line from the left bottom to the top right corner. (3) We calculated the model’s brier score, which is the mean squared difference between the predicted probability and the actual outcome and takes on a value between 0 and 1. The lower the brier score, the better the predictions are calibrated. Finally, we created an online hospitalisation risk estimator based on the final model (https://www.mornin-feng.com/all-projects-and-demos#gout).

Data processing and statistical analysis were carried out in Python 3.8 and R [10] using pandas [11], scikit-learn [12], and stats [10] libraries. Website development was done with Shiny library [13] in R. The Python and R codes used in this study can be found in our GitHub page (https://github.com/nus-mornin-lab/GoutAdmissionRiskEstimator).

## Results

**Fig. 1:**
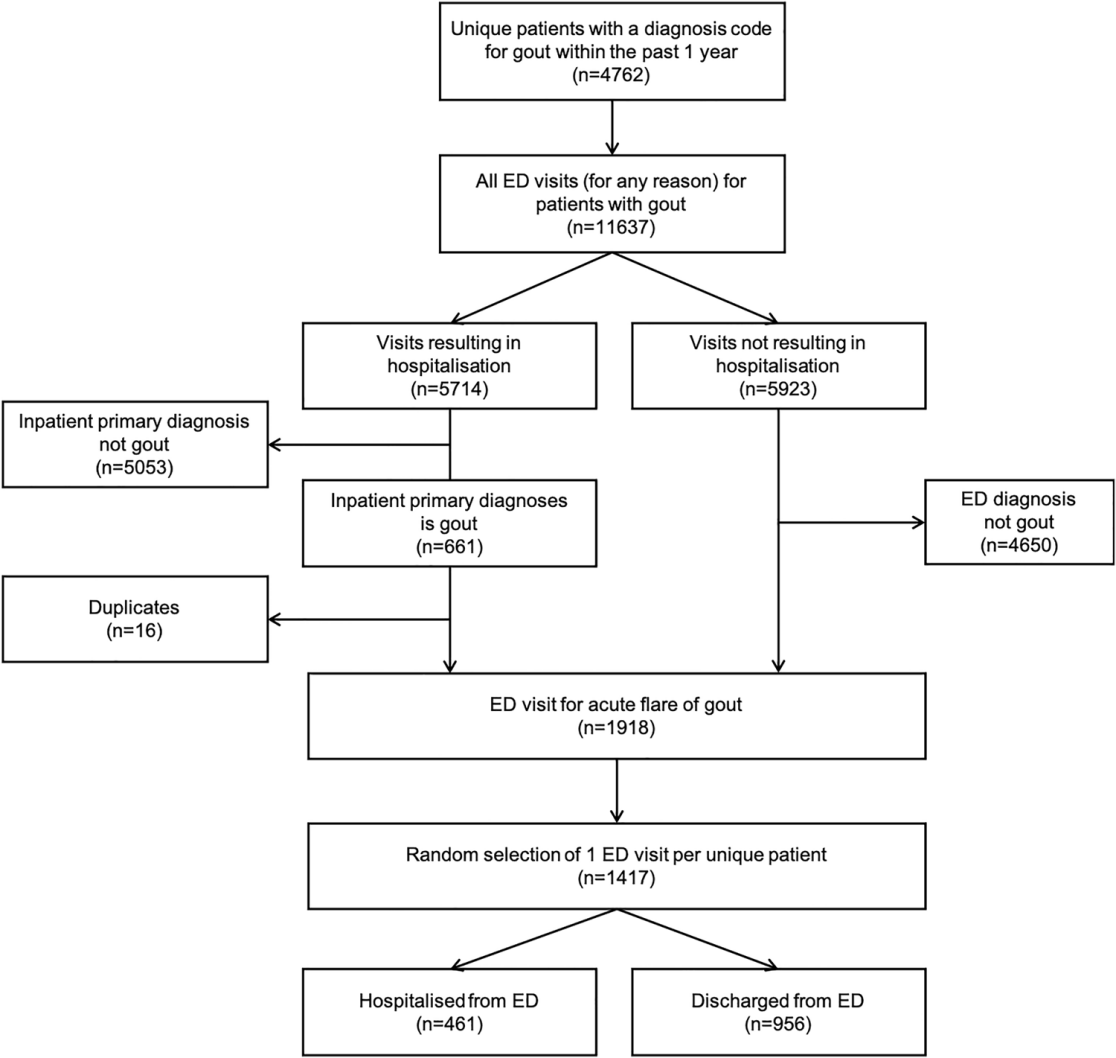
Illustration of the selection process performed to select the final cohort. From a dataset with 4762 unique patients and 11,637 ED cases, we included 1417 patients in our final cohort, each with 1 random ED visit chosen from all eligible visits

Figure 1 shows the dataset selection process. From a dataset of 4762 unique patients with a diagnosis code for gout recorded at any time between 1 January 2014 and 30 September 2017, we included 1918 ED attendances for gout between 1 January 2015 and 30 September 2017 among 1417 unique patients. Four hundred sixty-one (32.5%) of these ED attendances resulted in hospitalisation. Table 1 shows the baseline characteristics of the patients. The median (IQR) age was 56 [40, 70] years, and 1162 (82.0%) were males. Patients who were hospitalised were older (median [IQR] age 70 [59, 78] vs. 49 [35, 62]), less likely to be male (70.7% vs. 87.4%) and of different ethnic groups. All the comorbidity groups were seen more frequently in hospitalised patients. Prescription and dialysis codes and laboratory tests did not yield any additional patients with comorbidity over and above classification by diagnosis codes, testifying the robustness of the coding. Hospitalised patients were more likely to have a prescription code for ULT (19.3% vs. 10.0%) in the last 1 year. They were also more likely to have had a previous hospital stay with an inpatient primary diagnosis of gout (13.4% vs 2.3%) or a previous ED visit for gout (57.3% vs 37.8%). No significant difference was observed in the prevalence of previous outpatient visits for the management of gout and the prescription codes for AGT. Hospitalised patients were more likely to have a radiograph ordered for the upper limb joints (11.9% vs 7.7%) and for more joints (mean (SD) 0.8 (1.0) vs. 0.6 (0.8)). No significant difference was observed for whether any radiograph was ordered or whether a radiograph was ordered for the lower limb.

**Table 1:** Baseline characteristics of patients and comparison between those hospitalised vs discharged from the ED

Characteristics	Overall	Discharged	Hospitalised	*p*-value
*n* (%)	1417	956 (67.5)	461 (32.5)	
Demographics				
Age (median [Q1, Q3])	56 [40, 70]	49 [35, 62]	70 [59, 78]	** < 0.001**
Gender (male) (%)	1162 (82.0)	836 (87.4)	326 (70.7)	** < 0.001**
Race (%)				** < 0.001**
Chinese	759 (53.6)	485 (50.7)	274 (59.6)	
Malay	348 (24.6)	231 (24.2)	117 (25.4)	
Indian	110 (7.8)	79 (8.3)	31 (6.7)	
Others	200 (14.1)	161 (16.8)	39 (8.5)	
Comorbidities				
Hypertension (%)	463 (32.7)	166 (17.4)	297 (64.4)	** < 0.001**
Hyperlipidemia (%)	303 (21.4)	110 (11.5)	193 (41.9)	** < 0.001**
Cardiovascular disease (%)	226 (15.9)	75 (7.8)	151 (32.8)	** < 0.001**
Cancer (%)	50 (3.5)	17 (1.8)	33 (7.2)	** < 0.001**
Diabetes (%)	392 (27.7)	160 (16.7)	231 (50.1)	** < 0.001**
Chronic kidney disease (%)	354 (25.0)	118 (12.3)	235 (51.0)	** < 0.001**
Others (%)	126 (8.9)	57 (6.0)	69 (15.0)	** < 0.001**
Past medical resource utilisation (D-1 ~ D-365)				
Prescription for urate-lowering therapy (%)	212 (15.0)	96 (10.0)	89 (19.3)	** < 0.001**
Prescription for acute gout treatment (%)	363 (25.6)	243 (25.4)	120 (26.0)	0.855
Outpatient visits for gout (%)	116 (8.2)	74 (7.7)	42 (9.1)	0.437
Previous hospitalisation for primary diagnosis of gout (yes/no) (%)	79 (5.6)	22 (2.3)	62 (13.4)	** < 0.001**
Previous ED attendance (yes/no) (%)	628 (44.3)	361 (37.8)	264 (57.3)	** < 0.001**
Radiographs in the ED				
Had at least one radiograph (%)	706 (49.8)	463 (48.4)	243 (52.7)	0.146
On the lower limb^ (%)	610 (43.0)	403 (42.2)	207 (44.9)	0.357
On the upper limb^ (%)	129 (9.1)	74 (7.7)	55 (11.9)	**0.014**
Number of joints involved (mean (SD))	0.7 (0.8)	0.6 (0.8)	0.8 (1.0)	** < 0.001**

^Lower limb includes the ankle, knee, and foot; upper limb includes the hand, wrist, elbow, and shoulder

Table 2 shows the output from the multivariable logistic regression model. Older age and presence of hypertension and chronic kidney disease were associated with higher odds of hospitalisation. Previous hospitalisation for gout was also strongly associated with higher odds of hospitalisation (OR 4.80, 95% CI 2.34–9.85, *p* < 0.001). Prescription of AGT was associated with a lower odds of hospitalisation (OR 0.61, 95% CI 0.39–0.96, *p* < 0.05). The number of joints involved was associated with a higher odds of hospitalisation (OR 1.70, 95% CI 1.20–2.39, *p* < 0.01); however, it did not increase the performance of the model; hence, we excluded it from the final model. The coefficients for these variables as shown in Table 2 were used to build the risk estimator.

**Table 2:** Adjusted odds ratios, 95% confidence intervals, and coefficients for the odds of hospitalisation

Variable	OR	95% CI	*p*-value	Final coef
Intercept	0.02	[0.01, 0.06]	** < 0.001**	− 3.99
Age	1.04	[1.03, 1.05]	** < 0.001**	0.04
Race—Chinese (ref)				
Race—Indian	1.39	[0.94, 2.06]		
Race —Malay	1.06	[0.57, 1.97]		
Race—others	0.67	[0.39, 1.15]		
Gender—male	0.78	[0.53, 1.16]		
Hypertension	3.04	[2.00, 4.62]	** < 0.001**	1.22
Hyperlipidemia	1.20	[0.75, 1.92]		
Cardiovascular disease	1.32	[0.84, 2.08]		
Cancer	1.94	[0.88, 4.32]		
Diabetes	1.12	[0.72, 1.74]		
Chronic kidney disease	1.89	[1.25, 2.88]	** < 0.01**	0.77
Other comorbidities	0.97	[0.58, 1.64]		
Received urate-lowering therapy	1.02	[0.56, 1.86]		
Received acute gout treatment	0.61	[0.39, 0.96]	** < 0.05**	− 0.62
Had outpatient visits with gout diagnosis	0.62	[0.29, 1.30]		
Previous hospitalisation for gout	4.80	[2.34, 9.85]	** < 0.001**	1.39
Previous ED visits for gout	0.83	[0.57, 1.22]		
Had at least one radiograph	0.59	[0.18, 1.96]		
On any lower limb joints^	0.73	[0.23, 2.33]		
On any upper limb joints^	0.90	[0.25, 3.22]		
Number of joints involved	1.70	[1.20, 2.39]	** < 0.01**	

^Lower limb includes the ankle, knee, and foot; upper limb includes the hand, wrist, elbow, and shoulder

**Fig. 2:**
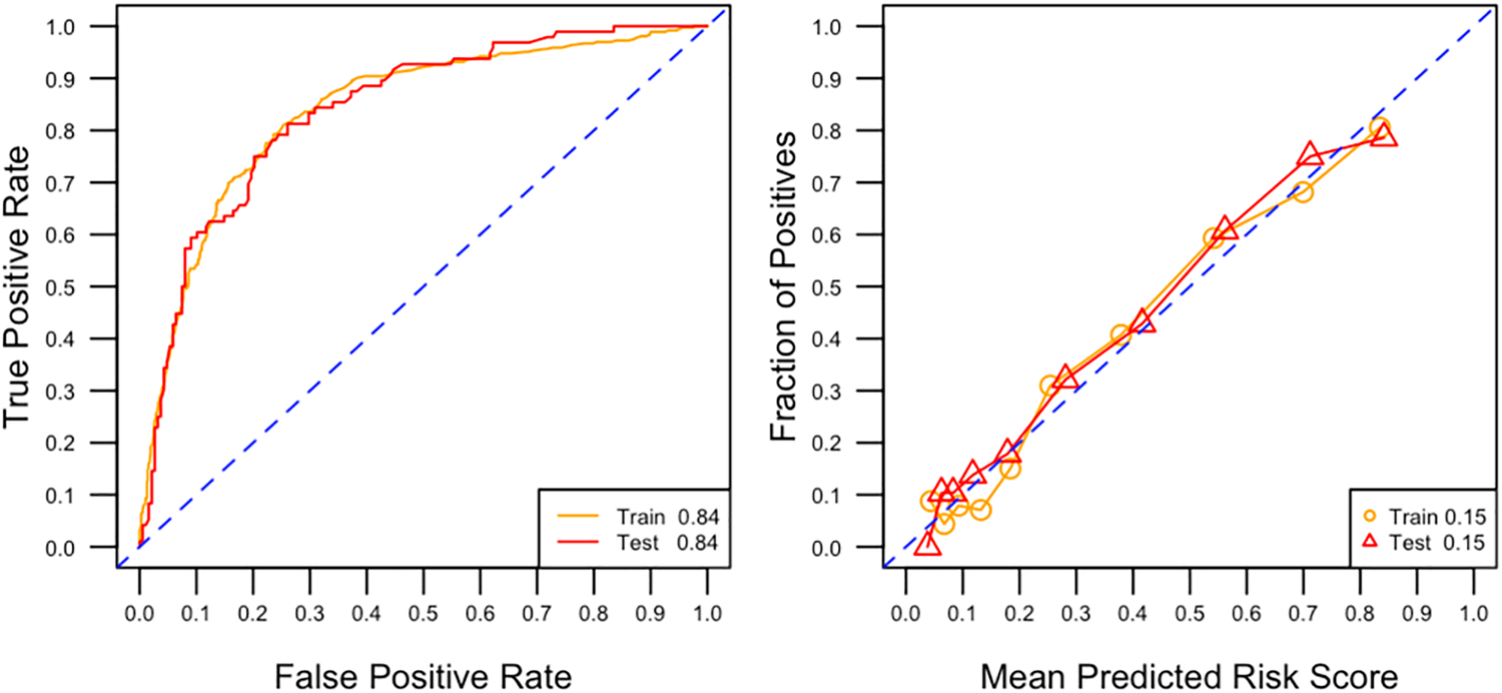
(Left) ROC curve of the final model on the derivation set and validation set. AUROC is reported in the legend. (Right) Calibration plot with 10 bins of the final model on the derivation set and validation set. Perfect calibration is represented by the diagonal dashed line. Brier score is reported in the legend

The ROC curve and the calibration plot of the final model are shown in Fig. 2. On the ROC curve, the model achieves an AUROC of 0.84 on both the training data and the test data. On the calibration plot, the model shows decent fit being close to the perfect diagonal calibration line. The brier scores are 0.15 on both the training data and the test data.

## Discussion

We have described a large dataset of patients with gout, and the factors associated with hospitalisation after an ED visit, and created an easy to use, web-based risk estimation tool, to estimate the risk of hospitalisation for patients with gout. We plan to apply our tool in outpatient or primary care settings to prioritise people at risk for hospitalisation for a higher level of resource allocation such as early specialist review, managed care, and/or more frequent review.

As expected, our study demonstrated a significant burden of comorbidity in patients hospitalised for gout; 64.4%, 41.9%, 32.8%, 50.1%, and 51.0% had hypertension, hyperlipidemia, cardiovascular disease, diabetes, and chronic kidney disease, respectively. While this is comparable with other studies, the proportion of patients hospitalised in our dataset was much higher than previously reported. 32.5% of our patients with gout were hospitalised compared to only 7.7% in the USA [7]; this may be possibly due to differences in our healthcare delivery and funding models and varying thresholds for hospitalisation. The presence of hypertension especially was associated with 3.04 times higher odds of gout-related hospitalisation. Previous hospitalisation for gout was associated with the highest odds of gout-related admission in our study, underscoring this as a marker of a cohort with higher healthcare needs. This may be indicative of suboptimal chronic disease management [14] and may identify a group of patients who would benefit from being enrolled into a chronic care management programme. The median of predicted risk scores is 0.60 for hospitalised patients versus 0.14 for discharged patients. Depending on the further external validation, we may choose, for example, a cut off score of 0.6 for a dichotomized recommendation of specialist care versus primary care.

The prescription of AGT was insignificant in the univariable analysis while significant in the multivariable analysis. Through further analysis, we found that the inclusion of AGT prescription increased the regression coefficients of previous hospitalisation for gout or the comorbidities. We suspect that prescription of AGT could be a suppressor variable [15] and will explore further in our subsequent studies. The analysis suggests that patients suffering from untreated gout flares were more likely to be hospitalised and treating flares with appropriate medications decreased hospitalisation for gout. Our univariable analysis demonstrated that the hospitalised group had a higher proportion of patients who received ULT in the past 1 year. We suspect that the association is likely confounded, as older gout patients with comorbidities tended to be followed up more often in our clinic for chronic diseases, as compared to younger and comparatively healthier gout patients who merely visited the ED for flare prescriptions and were less likely to be regularly reviewed. Our multivariable analysis also revealed that ULT was not a significant predictor of hospitalisation, confirming the confounding effect. We attempted to add a variable of the number of Charlson comorbidities into the model during our experiments, but we did not present it for the following 3 reasons: (1) it was highly correlated with our existing comorbidity variables, (2) it did not increase the performance of the model, and (3) it is not as easy to collect as our current variables, as the physician may not have access to all the comorbidities of the patient, especially in the primary care setting.

Our study has several strengths. We identified all patients with gout in our dataset over nearly 3 years, using all ICD-9 and ICD-10 diagnosis codes within a 1-year look-back period and further used prescription, dialysis, and laboratory data to ensure comprehensive inclusion of all cases. We only included 1 visit per patient (chosen randomly), to ensure all data were independent of each other. We used easily available clinical and demographic variables, allowing the resultant tool to be usable in an outpatient or primary care setting. We chose not to use ED laboratory values in our model because the ordering of laboratory tests may in itself be tied to the decision to admit a patient. Additionally, including laboratory values may decrease the applicability of the model in the outpatient and primary care setting. We split our dataset into a derivation and a validation cohort and tested the performance of our model using 3 different methods. Our resulting model with only 5 variables is simple, convenient to use, and yet has a high AUROC for predicting the likelihood of hospitalisation for patients after an ED visit for a gout flare. Finally, we developed a freely accessible web-based tool based on the model for potential users and open sourced the code of our study for better reproducibility.

Our study has certain limitations due to its retrospective nature. There may be misclassification of the diagnosis of a gout flare, especially in the patients who were discharged from ED, as it was mainly based on clinical diagnosis by the ED physicians. Additionally, our risk estimation tool is derived from a single centre and from patients who presented to the ED for gout flares, so further validation studies will be required before applying the tool to outpatient or primary care settings, especially in other populations. However, our observations of older age and prior hospitalisation as predictors of hospitalisation are consistent with another local retrospective study [16] investigating the impact of comorbidities, acute illness burden, and social determinants of health on the risk of hospital readmissions, and the characteristics and outcomes of our cohort are additionally similar to those described in other developed countries [2, 7]. We were unable to study other potential predictors of hospitalisation such as a higher tophaceous burden or erosive disease as these variables were unavailable in our administrative database. Data on severity of the gout flare and number or site of joints involved were similarly unavailable; however, we attempted by creating surrogate markers using the radiograph orders. After we conducted the analysis, none of them were included in the final model. We acknowledge that the decision for hospitalisation, made by the ED clinician, is an individualised decision. Non-clinical factors such as socioeconomic status, social condition and social isolation, functional limitation and pain control, post-ED discharge support, and acceptability of the hospitalisation would all contribute to the decision, which we were not able to address using our data. Nevertheless, our model’s performance was excellent as tested robustly using three different methods.

Our observations have important clinical implications for patients with gout and their physicians. Early identification of the patients with a high likelihood of gout-related hospitalisation using our web-based risk estimator may assist to target resources to the highest risk individuals. Eventually we hope this would lead to improved patient care and optimisation of healthcare utilisation.

## Supplementary Information

ESM 1 (DOCX 18.3 kb)

## Data Availability

The data is not publicly available due to the institution’s policy.

## Code availability

https://github.com/nus-mornin-lab/GoutAdmissionRiskEstimator
