# Supplementary material for: Hospital admission risk stratification of patients with gout presenting to the emergency department
Source: Clin Rheumatol. 2022 Jan 1;41(6):1801–7. doi: 10.1007/s10067-021-05902-5 (PMC9119890; doi:10.1007/s10067-021-05902-5)
Supplement: Supplementary file 1 — (DOCX 18.3 kb) [file 10067_2021_5902_MOESM1_ESM.docx]

**Supplementary Materials**

Supplementary Table 1: List of comorbidities included in our analysis. Comorbidity in the Charlson Comorbidity Index were selectively grouped. HIV was not included due to a prevalence of zero in our cohort.

| **Comorbidities Group** | **Charlson Comorbidities** |
| --- | --- |
| Hypertension | N.A. |
| Hyperlipidemia | N.A. |
| Cardiovascular Disease | Acute myocardial infarction |
|  | Congestive heart failure |
|  | Cerebral vascular accident |
|  | Peripheral vascular disease |
| Cancer | Cancer |
|  | Metastatic cancer |
| Diabetes | Diabetes |
|  | Diabetes complications |
| Chronic Kidney Disease | Renal Disease |
| Others | Connective tissue disorder |
|  | Dementia |
|  | Liver disease |
|  | Peptic ulcer |
|  | Pulmonary disease |
|  | Severe liver disease |
|  | Paraplegia |
